# Risk factors and spatial distribution of extended spectrum β-lactamase-producing- *Escherichia coli* at retail poultry meat markets in Malaysia: a cross-sectional study

**DOI:** 10.1186/s12889-016-3377-2

**Published:** 2016-08-02

**Authors:** A. B. Aliyu, A. A. Saleha, A. Jalila, Z. Zunita

**Affiliations:** 1 Department of Veterinary Pathology and Microbiology, Faculty of Veterinary Medicine, University Putra Malaysia, 43400 Serdang, Selangor Malaysia; 2 Department of Veterinary Clinical Studies, Faculty of Veterinary Medicine, University Putra Malaysia, 43400 Serdang, Selangor Malaysia; 3 Department of Pathology and Microbiology, Faculty of Veterinary Medicine, Veterinary Public health laboratory, Universiti Putra Malaysia, 43400 Serdang, Selangor Malaysia

**Keywords:** ESBL- *E. coli*, Zoonosis, Foodborne infection, Antimicrobial resistance, Poultry meat, Wet-market, Risk factor, Malaysia

## Abstract

**Background:**

The significant role of retail poultry meat as an important exposure pathway for the acquisition and transmission of extended spectrum β-lactamase-producing *Escherichia coli* (ESBL-EC) into the human population warrants understanding concerning those operational practices associated with dissemination of ESBL-EC in poultry meat retailing. Hence, the objective of this study was to determine the prevalence, spatial distribution and potential risk factors associated with the dissemination of ESBL-EC in poultry meat retail at wet-markets in Selangor, Malaysia.

**Methods:**

Poultry meat (breast, wing, thigh, and keel) as well as the contact surfaces of weighing scales and cutting boards were sampled to detect ESBL-EC by using culture and disk combination methods and polymerase chain reaction assays. Besides, questionnaire was used to obtain data and information pertaining to those operational practices that may possibly explain the occurrence of ESBL-EC. The data were analysed using logistic regression analysis at 95 % CI.

**Results:**

The overall prevalence of ESBL-EC was 48.8 % (95 % CI, 42 – 55 %). Among the risk factors that were explored, type of countertop, sanitation of the stall environment, source of cleaning water, and type of cutting board were found to be significantly associated with the presence of ESBL-EC.

**Conclusions:**

Thus, in order to prevent or reduce the presence of ESBL-EC and other contaminants at the retail-outlet, there is a need to design a process control system based on the current prevailing practices in order to reduce cross contamination, as well as to improve food safety and consumer health.

**Electronic supplementary material:**

The online version of this article (doi:10.1186/s12889-016-3377-2) contains supplementary material, which is available to authorized users.

## Background

Poultry meat constitutes a greater percentage of human protein sources; however, it may also serve as an important medium for the transfer of multidrug-resistant bacteria, such as extended spectrum β-lactamase-producing *Escherichia coli* (ESBL-EC), from food-producing animals to the consumers. ESBL-EC is an emerging zoonotic and a multidrug-resistant bacterium, which has currently posed a major challenge in antimicrobial treatments [1]. Antimicrobial resistance and food safety in recent years, have received global attention over their huge impact on population health and global economy [2].

Besides, molecular epidemiological studies have shown that large proportions of resistant *E. coli* causing blood stream infections within human population are of food animal origin [3, 4]. According to WHO global report on antimicrobial resistance [2], ESBL-EC is among the most frequent causes of blood stream infection, community and hospital acquired urinary tract infection, also with the associated increase in mortality rate and cost of hospitalization, prolonged hospital stay, as well as the world most leading cause of foodborne infection. Foodborne infection with ESBL-EC, moreover, has been associated with limited therapeutic options, prolong duration of infection, high rate of treatment failure with increased morbidity, and mortality rate [5, 6].

Furthermore, several studies have consistently revealed the role of retail poultry meat in the transmission of multidrug resistant bacteria among human population and their environment, resulting in increased challenge to food safety and environmental health [7]. Other than that horizontal transfer of ESBL-EC has been believed to be the major pathway that fosters its global dissemination [8] through improper handling of poultry during slaughtering, dressing and other meat processing operations as well as at retailing.

Thus, ESBL-EC has been reported as a threatening issue in the Malaysian healthcare setting [9, 10], in addition to several research reports demonstrating the potential role of retail poultry meat as a major reservoir for human exposure to ESBL-EC [7, 11, 12]. However, little or no study has been conducted to study its prevalence and the risk factors that may foster its dissemination at retail poultry meat-markets in Malaysia. Hence, the objective of this study was to determine the prevalence and risk factors associated with ESBL-EC at retail poultry meat wet-markets in Selangor, Malaysia.

## Methods

### Study area

Selangor is one of the 13 states of Malaysia; with a total area of 8,104 km^2^, the Malaysia’s most populous state, also known as the industrial hub of Malaysia, with the largest economy in terms of gross domestic product (GDP), providing almost 23 % of Malaysian GDP; it is located at centre of Peninsular Malaysia [13–15]. Almost 90 % of Malaysian poultry production is in peninsular Malaysia (while the remaining is in East Malaysia), which was reported to be among the world highest per capita consumption of poultry meat at 40 kg per year [16]. Poultry meat is the most stable protein source across all ethnic groups of the Malaysian population [16]. According to the USDA recent International Egg and Poultry review [17], Malaysia is self-sufficient in poultry meat production, which was almost entirely of broiler meat, with production forecast at 1.44 million tons; about 40 % are marketed to the consumers through wet-markets [17].

### Collection of sample

A total of 240 samples comprising 160 broiler chicken meat samples (breast, wing, thigh, and keel) and 80 swab samples from contact surfaces (weighing scales and cutting boards/instrument) were examined to detect the presence of ESBL-EC. The estimated sample size was calculated using G*Power 3.1.9.2, using A priori F-tests ANOVA: Fixed effects, omnibus, and one-way; given α = 0.05, Power = 0.80, Effect size = 0.25, Number of groups = 8. Informed consents were obtained from each participant prior to sample collection and questionnaire administration. The participants were informed about the purpose of the research, likewise they were assured of protecting their identities and received information would be highly confidential, as it was only used for research purpose.

A total number of 40 individual stalls were selected at random within eight districts in Selangor (Table 2). Three wet-markets were selected in every district, out of which five different stalls were recruited to represent a district area. At each stall, six representative samples were obtained, which consisted of four broiler chicken meats (breast, keel, wing and thigh) and two swabs (weighing scale and cutting board/instrument). The meat samples were bagged separately in a sterile plastic bag. The surfaces of the cutting board/instrument and weighing scale covering an area of 25 cm^2^ were swabbed using pre-moistened sterile cotton swab, [18], and each swab was placed in sterile transport media containing 9 ml of peptone water (Oxoid). All samples were transported to the laboratory in ice cool box and processed within five hours of collection.

### Sample preparation

Each meat sample was processed by taking 25 g of the meat sample, vigorously shaken in a sterile plastic bag containing 225 ml of peptone water and homogenized in a stomacher for 1 min [19]. Five ml of each homogenized sample was subsequently placed in 45 ml of peptone water and vortexed gently to form a mixture of processed meat sample. Each of the bottles containing the swab was vortexed gently; then, 1 ml was taken and diluted in 9 ml of peptone water and was further vortexed gently.

### *E. coli* isolation and identification

A few loopfulls of each processed sample was directly inoculated on Chromocult Coliform agar (Merck, UK) and incubated at 37 °C for 24 h. The agar served as differential and selective media, it distinguished *E. coli* from other coliform colonies and inhibited gram-positive bacteria growth, which was further confirmed by using Kovac’s reagent (the blue-violet *E. coli* colony becomes red) as indicated by the manufacturer.

### Screening for extended spectrum β-lactamases production

The non-duplicate representative of *E. coli* isolates were subcultured on CHROMagar ESBL (CHROMagar, Paris, France) to screen for the presence of ESBLs [20]. The plates were incubated aerobically overnight at 37 °C. The presumptively identified ESBL-EC colonies were selected (dark pink to reddish colonies) and further subjected to phenotypic confirmation.

### Phenotypic confirmation of extended spectrum β-lactamases production

The phenotypic confirmation was conducted by using the Combination Disk Method, as described by CLSI [21]. The bacterial suspension (turbidity of 0.5 McFaland standard) was spread on Mueller-Hinton agar (oxoid) impregnated with cefotaxime (CTX-30 μg), ceftazidime (CAZ-30 μg), cefotaxime/clavulanic acid (CTX/CV), and ceftazidime/clavulanic acid (CAZ/CV) disks, and incubated at 37 °C for 16 to 18 h. The interpretation was based on the zones of inhibition produced by cefotaxime (CTX-30 μg) and ceftazidime (CAZ-30 μg) disks against cefotaxime/clavulanic acid (CTX/CV), and ceftazidime/clavulanic acid (CAZ/CV) respectively [21]. A difference of ≥5 mm between the zones of CTX and CTX/CV or CAZ and CAZ/CV was considered as phenotypically confirmed ESBLs. *Klebsiella pneumoniae* ATCC 700603 and *Escherichia coli* ATCC 25922 were used as quality control strains [21].

### DNA extraction and PCR amplification

The DNA of phenotypically confirmed ESBL-EC was isolated by using the boiling method, on 2–3 colonies grown on nutrient agar. The colonies were suspended in 100 μl of sterile water, and the suspensions were boiled for 15 min, cooled to 4 °C, and then, subsequently centrifuged for 30 s at 12 000 × g. The supernatant served as a source of DNA template for the PCR, while the specific oligonucleotide primers for *bla*_TEM_, *bla*_SHV_, *bla*_CTX-M_, *bla*_OXA,_ and *E. coli* were used for amplifications of these *bla*-genes and *E. coli*. Furthermore, MyTaq Protocol (BIOLINE) was used as standard for the PCR reactions and amplification. The sizes of *bla*_CTX-M_, *bla*_TEM_, *bla*_SHV_, *bla*_OXA,_ and *E. coli* amplicons are shown in Table 1, using *K. pneumoniae* ATCC 700603 and EC1003-1 as positive controls. The amplified products were separated by gel electrophoresis on 1.5 % agarose gels stained with ethidium bromide.

**Table 1:** PCR primers used for the detection β -lactamase encoding genes

Target gene	Primer	Sequence (5′ – 3′)	Size of product (bp)	GenBank accession no
*bla*_SHV_	Forward	CAATCACGACGGCGGAATCT	168	AB731686
	Reverse	GTGGGTCATGTCGGTACCAT		
*bla*_CTX-M_	Forward	AAGCACGTCAATGGGACGAT	402	JN411912
	Reverse	GTTGGTGGTGCCATAGCCA		
*bla*_TEM_	Forward	TCCTTGAGAGTTTTCGCCCC	643	EU352903
	Reverse	TGACTCCCCGTCGTGTAGAT		
*bla*_OXA_	Forward	TTGCACTTGATAGTGGTGTGA	250	JN003412
	Reverse	AGTGAGTTGTCAAGCCAAAAAGT		
*E. coli*	Forward	TGACGTTACCCGCAGAAGAA	832	X80724
	Reverse	CTCCAATCCGGACTACGACG		

### Questionnaire design and definition of terms

A questionnaire was developed as an instrument to gather information and data for the risk factors analysis. The items were derived from literature search, interviews, and focus group discussions with local veterinarians and representative of butchers. The questionnaire was further evaluated by the research team for face validation. In order to evaluate the adequacy of the instrument content, the questionnaire was further assessed by five experts in the field for content validity. On top of that, the instrument was refined and pilot tested. Unclear items were finally dropped, while items with overlapping meaning were merged together as single items.

#### Sanitation of the stall environment

Stall sanitation is classified into three hygiene categories, namely good, fair, and poor. A stall with clean floor, clean countertop, and absence of flies is considered as good; while those with either clean floor/clean counter top, in the absence of flies is considered as fair hygiene; and those with only one of the three characteristics is considered as poor hygiene.

#### Type of countertop

Refers to the type of material used as benchtop/working-surface, as there are four types of countertop, namely wooden, tile, plastic-sheet, and stainless-steel.

#### Source of cleaning water

Butchers mainly obtain water either directly from running-tap or from a water container, which they use for washing hands, as well as sanitising utensils, equipment, and other contact surfaces throughout the meat selling/processing period.

#### Type of cutting board/instrument

Refers to the type of material used by butchers as chopping-block. Two types of cutting boards were identified, namely rubberwood (wooden), polyethylene cutting board (plastic); and the cutting instrument was an electric meat cutter made of stainless steel.

### Statistical analysis

Data were analysed by using SPSS version 20. chi-square test was used to compare the prevalence; whereas the relationship between potential risk factors and detection of ESBL-EC was explored by using univariate logistic regression at 95 % confidence interval (95 % CI). However, only factors that were found to be significantly associated with detection of ESBL-EC had been included in the multivariate logistic regression model for estimation of their odds ratio at 95 % CI.

## Results

### Spatial distribution of ESBL-EC

**Fig. 1:**
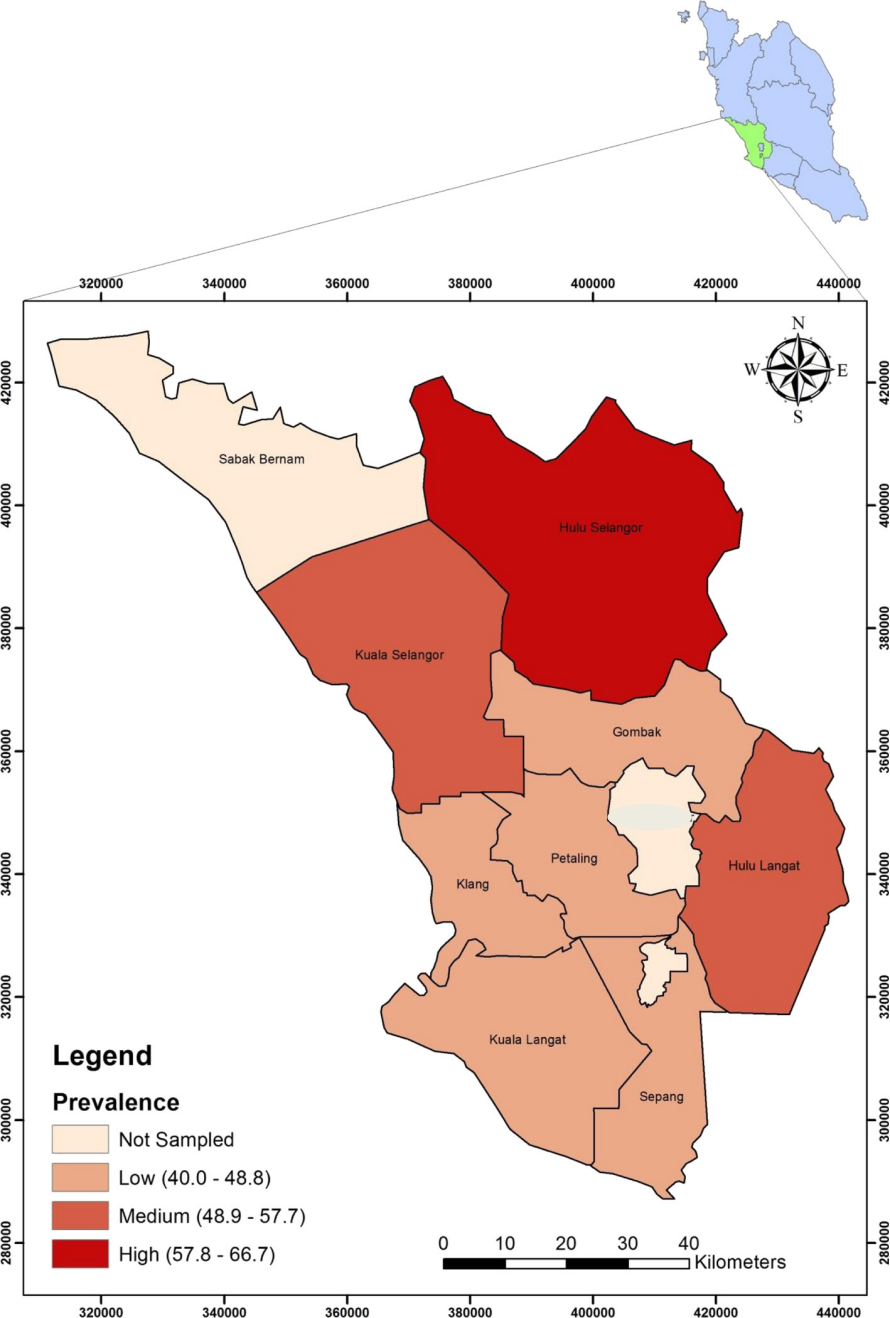
Choropleth map of spatial distribution of ESBL-EC in Selangor, Malaysia

A total of 240 samples were collected in wet-markets within eight districts of Selangor from July −2012 to February 2013. The overall prevalence of ESBL-EC was 48.8 % (95%CI, 42 – 55 %), although the difference in prevalence was not statistically significant between the districts (*χ*2 = 6.921, *df* = 7, p = .437) with chi-square test at 95 % confidence interval (95 % CI). Stalls in the district of Hulu Selangor were shown to have the highest prevalence at 66.7 %. while moderate prevalence was observed in Hulu Langat at 56.7 % and Kuala Selangor at 50 %. Nevertheless, the lowest prevalence was found in Klang at 46.7 %, Sepang at 46.7 %, Petaling at 43.3 %, Gombak at 40 %, and Kuala Langat at 40 % (Fig. 1; Table 2).

**Table 2:** Prevalence of ESBL-EC at retail poultry meat wet-markets

District area	No. of samples	No. of positive samples	Prevalence %
Hulu Selangor	30	20	66.7
Hulu langat	30	17	56.7
Kuala Selangor	30	15	50.0
Klang	30	14	46.7
Sepang	30	14	46.7
Petaling	30	13	43.3
Gombak	30	12	40.0
Kuala Langat	30	12	40.0
Total ESBL	240	117	48.8

**Fig. 2:**
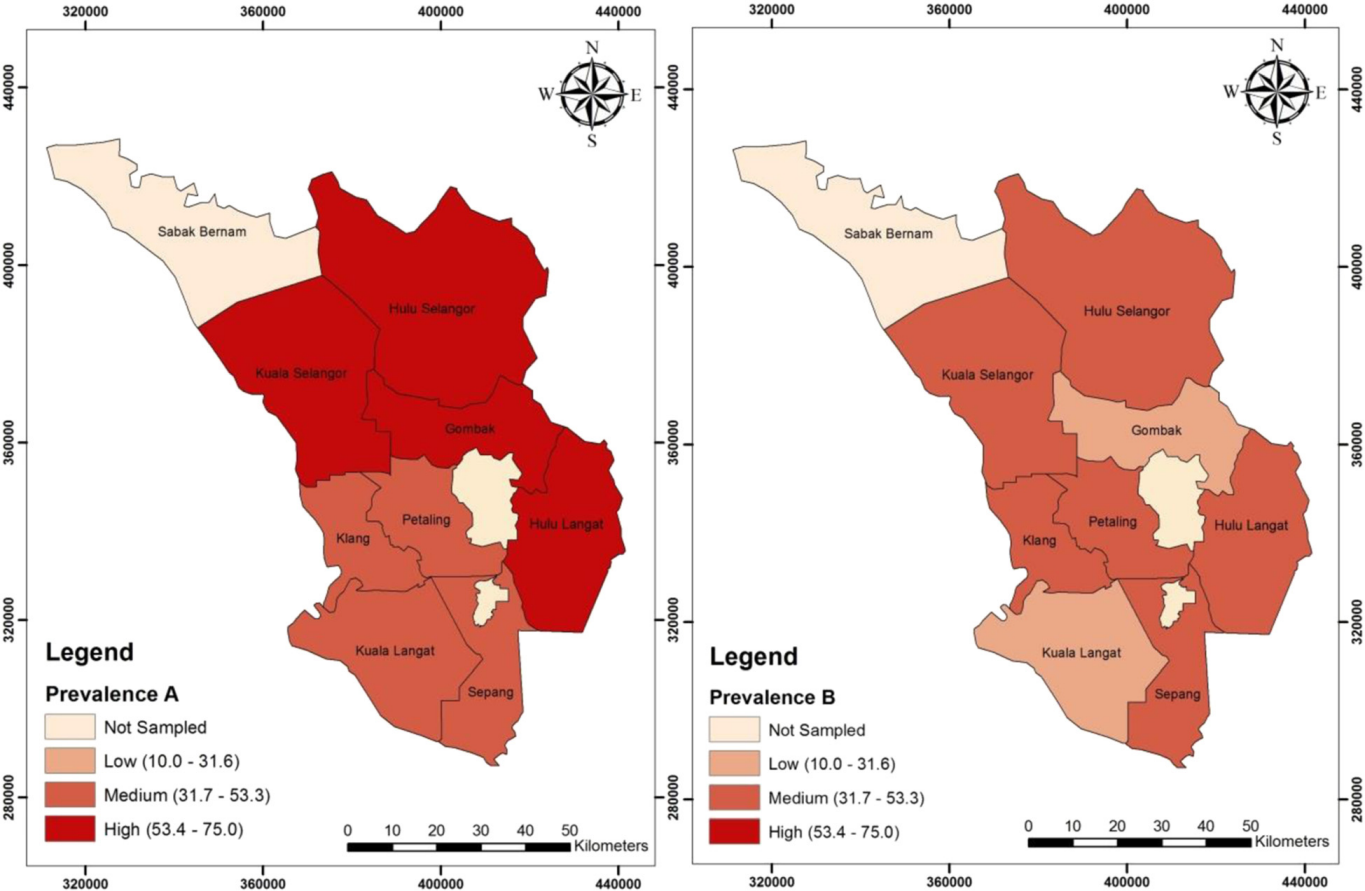
Choropleth map of variation in spatial distribution of ESBL-EC between meat (**a**) and contact surfaces (**b**) in Selangor, Malaysia

However, when the occurrence of ESBL-EC in meat was compared to that of the contact surfaces, the meat samples displayed higher occurrence rate of 53.8 % compared to contact surfaces at 38.8 % (Fig. 2; Table 3).

**Table 3:** Variation in the prevalence of ESBL- EC between meat and its contact surfaces

District area	ESBL- EC in poultry meat		ESBL- EC in contact surfaces	
	No. of samples	Prevalence %	No. of samples	Prevalence %
Hulu Selangor	20	75.0	10	50.0
Hulu Langat	20	65.0	10	40.0
Gombak	20	55.0	10	10.0
Kuala Selangor	20	55.0	10	40.0
Klang	20	50.0	10	40.0
Kuala Langat	20	45.0	10	30.0
Sepang	20	45.0	10	50.0
Petaling	20	40.0	10	50.0
Total ESBL	160	53.8	80	38.8

Furthermore, more than 50 % of the meats sampled were contaminated with ESBL-EC; the highest occurrence was in breast, wing, and thigh with proportions of 65, 52, and 50 % respectively. Keel and weighing scale had moderate rates, while cutting board/instrument had rather lower rates at 47.5, 40, and 37.5 % respectively (Table 4).

**Table 4:** Occurrence of ESBL- EC within samples collected

Source of sample	No of samples	No. of positive samples	Proportion of positive (%)	95 % CI	
				Lower	Upper
Breast	40	26	65.0	50	80
Wing	40	21	52.5	36	69
Thigh	40	20	50.0	34	66
Keel	40	19	47.5	31	64
Weighing scale	40	16	40.0	24	56
Cutting board	40	15	37.5	22	53
Total	240	117	48.8	42	55

### Risk factors associated with ESBL-EC contamination

Univariable logistic regression model indicated four factors that had the likelihood of causing ESBL-EC contamination, at *p*-value less than 0.05, which included stall sanitation, type of counter top, source of cleaning water, and type of cutting board/instrument (Table 5).

**Table 5:** Univariable and multivariable factors associated with ESBL-EC at retail poultry meat wet-markets

Variables	Univariable analysis		Multivariable analysis	
	OR^a^	95 % CI^b^	OR^a^	95 % CI^b^
Stall sanitation				
Poor	6.044	3.007–12.148**	3.122	1.319–7.391*
Fair	2.346	1.154–4.770*		
Good	1.00	Ref		
Type of counter top				
Wooden counter	8.125	2.509–26.311**	3.789	1.045–13.741*
Tiles counter	4.212	2.134–8.314**		
Plastic sheet	3.693	1.660–8.216**		
Stainless steel counter	1.00	Ref		
Source of cleaning water				
Container water	3.171	1.212–8.297*		
Tap water	1.00	Ref		
Type of cutting board/instrument				
Wooden	5.500	2.049–14.763*		
Plastic	2.419	1.015–5.763*		
Stainless steel cutter	1.00	Ref		
Wearing working attire				
No	1.352	.417–4.384		
Yes	1.00	Ref		
Butchers sanitation				
Poor	2.000	.354–11.296		
Fair	1.849	.324–10.548		
Good	1.00	Ref		
Use of PPE				
Poor	1.400	.403–4.862		
Fair	1.333	.406–4.373		
Good	1.00	Ref		

Ref = Reference variable

*p-value less than 0.05, **p-value less than 0.001

^a^ Odds Ratio

^b^ 95 % Confidence interval

However, based on the multivariable logistic regression model, only two factors significantly predicted ESBL-EC contamination, which were stall sanitation, and type of countertop (Table 5).

## Discussion

The distribution of ESBLs has been found to be varied worldwide, with Asia having the highest prevalence [22]. Base on study for Monitoring Antimicrobial Resistance Trends (SMART) in 2007, Asia-Pacific Region reported highest prevalence of ESBL-EC in India, China, and Thailand at 79.0, 55.0, and 50.8 % respectively. Meanwhile, moderate distribution of ESBLs was discovered in Vietnam, Singapore, and South Korea, at 34.4, 33.3 and 22.7 % respectively, and the least from Hong Kong, Philippines, Taiwan, Australia, and New Zealand at 17.8, 17.0, 12.7, 7.7, and 3.2 % respectively [23]. In Malaysia, Hashim et al. [10] reported 73.7 % of ESBL- *K. pneumonia* and 8.8 % of ESBL-EC among drug resistant bacteria isolated from the largest Malaysian tertiary referral hospital [10].

Environment, workers, equipment, and contaminated chicken were reported to be the major sources of meat contamination at retail outlets mainly due to improper hygiene practices and poor environmental sanitation. These factors favour the survival, proliferation, dissemination, and cross-contamination of retail meat and its environment with microbial agents [24–26]. At retail stalls, raw and packaged chicken drips continue to seep around stall environment, hence cross-contaminating meat, countertop, other contact surfaces, as well as floor [26, 27], more so, it might increase chances of occupational exposure especially via accidental cuts from contaminated utensils. Studies also have shown that thorough cleaning and regular disinfecting meat processing environment serves as important factor in reducing the risk of cross-contamination within meat processing environment [28, 29].

The present study had investigated the role of environmental sanitation in the likelihood of the occurrence of ESBL-EC on the meat. At univariate logistic model, it was found that stalls with poor sanitary environment were 6 times more at risk of ESBL-EC contamination in comparison to those with good hygiene (OR 6.044, 95 % CI = 3.007–12.148), while those with fair hygiene had twice the risk in contrast to those with good hygiene environment (OR 2.346, 95 % CI = 1.154 – 4.770). Apart from the above mentioned cross-contamination factors that may favour the observed occurrence, most of these chicken meats were placed on countertop without being chilled, such as using ice. Thus, the warm condition favours the survival and the multiplication of ESBL-EC, which can remain infectious for a long period even under adverse environmental condition [27, 30].

In addition, working surface or countertop has also been shown to play an important role in the dissemination of microorganisms at processing and retailing outlets. The question is what type of countertop surface material poses greater risk to food in relation to cross-contamination? and this depends on the availability of both intrinsic and extrinsic factors that allow bacterial survival, growth, and proliferation on the surface material [31, 32]. At univariate logistic model, the current study found that vendors who used working surfaces or countertops made of wooden material had 8.1 times the risk of contamination with ESBL-EC compared to stainless steel (OR 8.125, 95 % CI = 2.509 – 26.311), while tiles countertops had 4.2 times the risk of ESBL-EC contamination than stainless steel (OR 4.212, 95 % CI = 2.134 – 8.314). Nonetheless, the plastic-sheet covered countertop showed to have 3.7 times more risk of contamination than stainless-steel (OR 3.693, 95 % CI = 1.660 – 8.216).

In fact, both cutting board and working surface/countertop were reported to share similar factors in terms of cross contamination liability; these factors included board materials, scoring (degree of roughness) on the cutting/contact surfaces and its level of contamination, type of pathogen, part of the chicken and meat temperature during retailing, meat pH level, and water activity [31, 33–35]. The present study also found that the cutting-board material played significant role in the occurrence of ESBL-EC when using stainless steel cutting-instrument as reference model for comparison. At univariate logistic model, the wooden cutting board was found to have the highest risk of ESBL-EC contamination, at 5.5 times the risk compared to stainless steel (OR 5.50, 95 % CI = 2.049 – 14.763), while plastic cutting board had twice the risk of contamination than the stainless steel cutting-instrument (OR 2.419, 95 % CI = 1.015 – 5.763).

Additionally, one of the unhygienic practices that was revealed worth reporting is the source of water for washing hands and cleaning utensil during retailing. Majority of the meat vendors are using the same water placed in a container to wash their hands and utensils throughout the retailing period, thus the contaminated water continued to contaminate subsequent meat, contact surfaces and processing environment. At univariate logistic model, the present study found that meat vendors who used the same contaminated water for cleaning purpose had thrice the risk of causing cross-contamination compared to those who used direct water from the tap (OR 3.171, 95 % CI = 1.212 – 8.297).

Apart from temporal ambiguity associated with cross-sectional study design, additional limitation of the current study is the lack of data from human sample. This may provide additional information to the observed phenomenon, although attempt has been made to collect rectal swabs from the butchers, but they were reluctant to volunteer.

## Conclusion

Identifying risk factors associated with various components of poultry meat retailing can provide a scientific basis for designing effective risk communication and management strategy. Hence, the results obtained from this study suggest that veterinary/public health intervention needs to focus on promoting good personal hygiene, water and stall sanitation; poster teaching about cross contamination, proper cleaning and disinfecting work surfaces; encouraging the use of stainless steel countertop and plastic cutting board or stainless-steel cutting instrument; and meat handlers need to be educated on the global burden of foodborne diseases as well as its impact upon trade and development.

## Abbreviations

ANOVA, analysis of variance; CLSI, clinical and laboratory standards institute; DNA, deoxyribonucleic acid; GDP, gross domestic product; PCR, polymerase chain reaction; SMART, study for monitoring antimicrobial resistance trends; USDA, United States department of agriculture; WHO, world health organization

## Additional file

Additional file 1: Logistic regression tables for the factors associated with ESBL-EC at retail poultry meat wet-market. (DOCX 17 kb)
