# Supplementary material for: Risk factors and spatial distribution of extended spectrum β-lactamase-producing- Escherichia coli at retail poultry meat markets in Malaysia: a cross-sectional study
Source: BMC Public Health. 2016 Aug 2;16:699. doi: 10.1186/s12889-016-3377-2 (PMC4971674; doi:10.1186/s12889-016-3377-2)
Supplement: Additional file 1: — Logistic regression tables for the factors associated with ESBL-EC at retail poultry meat wet-market. (DOCX 17 kb) [file 12889_2016_3377_MOESM1_ESM.docx]

Univariable factors associated with ESBL-EC at retail poultry meat wet-markets

| Variables | Odds ratio | 95% confidence interval | | p-value |
| --- | --- | --- | --- | --- |
|  |  | Lower | Upper |  |
| **Stall sanitation**  Poor  Fair  Good | 6.044  2.346  Ref | 3.007  1.154 | 12.148  4.770 | **>.001***  >.001  .018 |
| **Type of counter top**  Wooden counter  Tiles counter  Plastic sheet  Stainless steel counter | 8.125  4.212  3.693  Ref | 2.509  2.134  1.660 | 26.311  8.314  8.216 | **>.001***  >.001  >.001  .001 |
| **Source of cleaning water**  Container water  Tap water | 3.171  Ref | 1.212 | 8.297 | **.019***  .019 |
| **Type of cutting board/instrument**  Wooden  Plastic  Stainless steel cutter | 5.500  2.419  Ref | 2.049  1.015 | 14.763  5.763 | **.002***    .001  .046 |
| **Wearing working attire**  No  Yes | 1.352  Ref | .417 | 4.384 | **.616**  .616 |
| **Butchers sanitation**  Poor  Fair  Good | 2.000  1.849  Ref | .354  .324 | 11.296  10.548 | **.721**  .433  .489 |
| **Use of PPE**  Poor  Fair  Good | 1.400  1.333  Ref | .403  .406 | 4.862  4.373 | **.869**  .596  .636 |

p-value significant at 0.05, *= Significant p-value, Ref= Reference variable

Multivariable factors associated with ESBL-EC at retail poultry meat wet-markets

| Variables | Odds ratio | 95% confidence interval | | p-value |
| --- | --- | --- | --- | --- |
|  |  | Lower | Upper |  |
| **Stall sanitation**  Poor  Fair  Good | 3.122  1.518  Ref | 1.319  .688 | 7.391  3.348 | **.029***  .010  .301 |
| **Type of counter top**  Wooden counter  Tiles counter  Plastic sheet  Stainless steel counter | 3.789  1.971  2.053  Ref | 1.045  .857  .825 | 13.741  4.533  5.106 | **.170**  .043*  .110  .122 |
| **Source of cleaning water**  Container water  Tap water | 1.570  Ref | .474 | 5.208 | .**461**  .461 |
| **Type of cutting board/instrument**  Wooden  Plastic  Stainless steel cutter | 1.981  1.517  Ref | .549  .471 | 7.151  4.885 | **.569**  .297  .485 |

p-value significant at 0.05, *= Significant p-value, Ref= Reference variable
